# Supplementary material for: Regulation of Rad52-dependent replication fork recovery through serine ADP-ribosylation of PolD3
Source: Nat Commun. 2023 Jul 18;14:4310. doi: 10.1038/s41467-023-40071-w (PMC10354178; doi:10.1038/s41467-023-40071-w)
Supplement: Supplementary file 1 — Supplementary Information [file 41467_2023_40071_MOESM1_ESM.pdf]

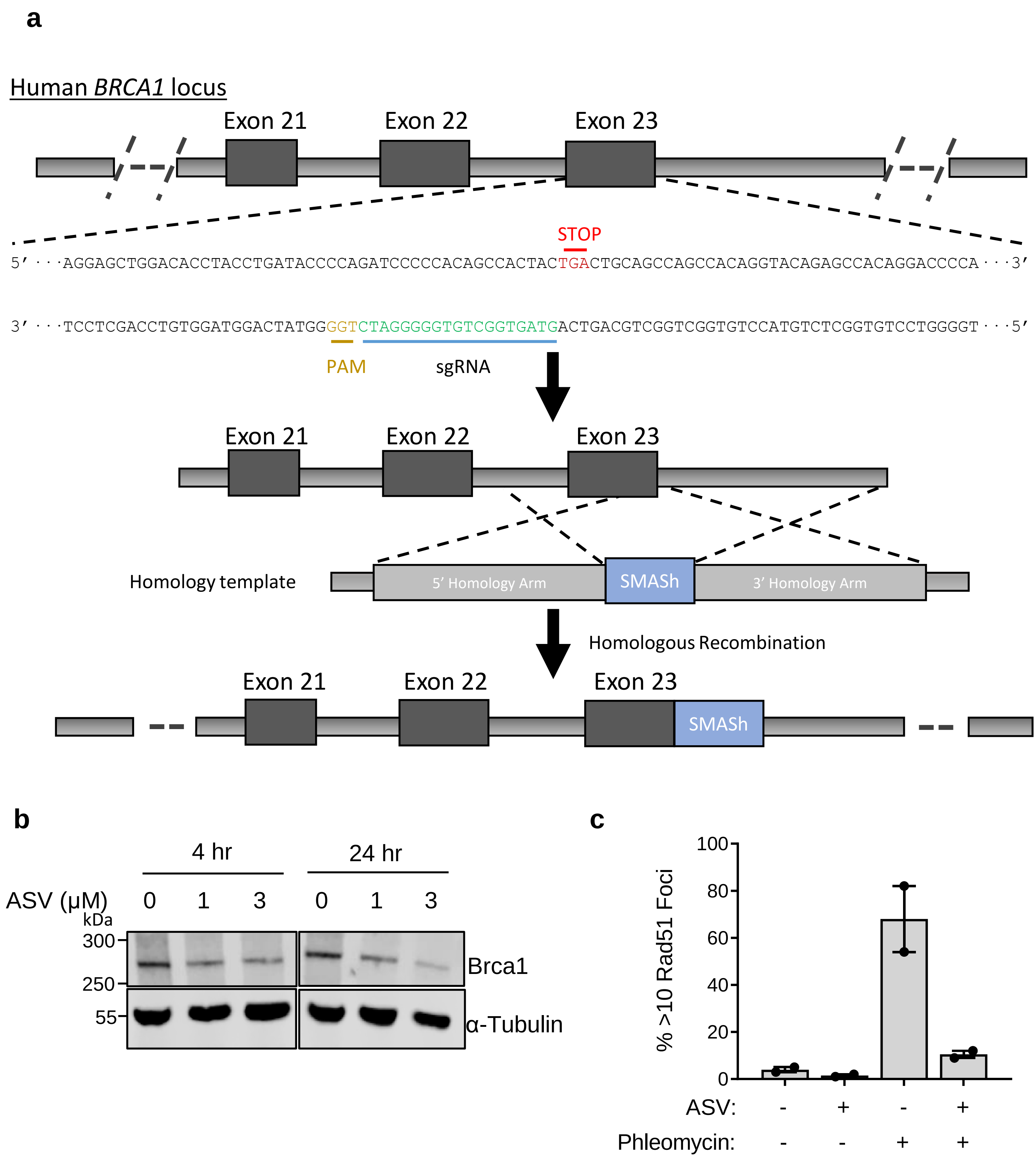

Supplementary Figure 1: Generation of *BRCA1*^{SMASh} cell lines

a. CRISPR-cas9 gRNA was designed adjacent to the stop codon of *BRCA1*, where the SMASh tag was to be inserted. Template DNA containing the SMASh sequence and flanking homology arms was transfected alongside the gRNA is indicated.

b. Inducible depletion of BRCA1-SMASh detected in nuclear cell extracts from BRCA1^{SMASh} cells treated with DMSO, 1 or 3 µM ASV for the indicated times. Data are representative of 3 independent experiments.

c. Analysis of DSB-induced Rad51 foci in *BRCA1*^{SMASh} cells. Cells were cultured in the presence or absence of 3µM ASV as indicated to deplete BRCA1-SMASh. Cells were left untreated or exposed to phleomycin for 1 hour and Rad51 nuclear foci detected by immunofluorescence. Data are presented as mean values and error bars represent +/- SEM of four independent experiments.

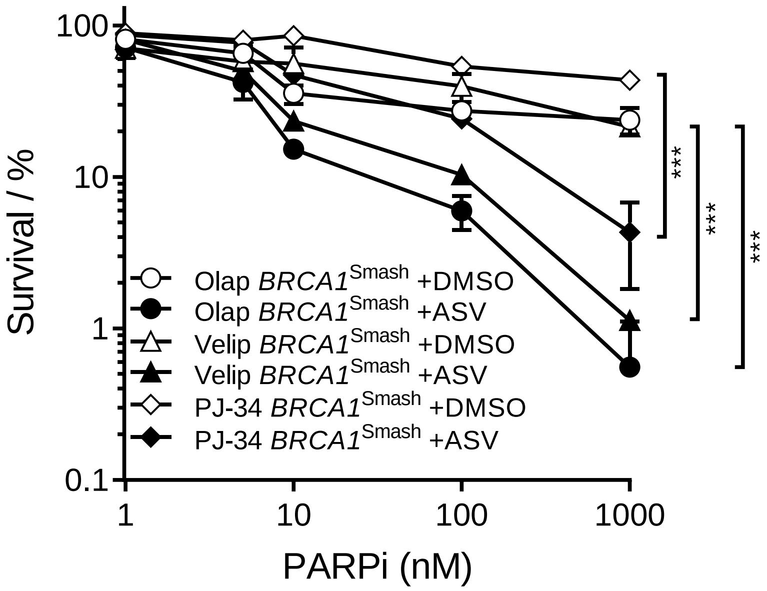

Supplementary Figure 2: Depletion of BRCA1^{SMASh} sensitises cells to a variety of PARP inhibitors. Clonogenic survival assay assessing sensitivity of *BRCA1*^{SMASh} cells to the PARPi olaparib (olap), veliparib (velip) and PJ-34 following depletion of BRCA1-SMASh using 3µM ASV. Statistical analysis was performed using a two-tailed Student's t test comparing survival at 1000 nM PARPi. Data are presented as mean values and error bars represent +/- SEM of three independent experiments. Statistical significance was calculated to a confidence of 95%, p<0.05 (*), 99%, p<0.01 (**) or 99.9%, p<0.001 (***).

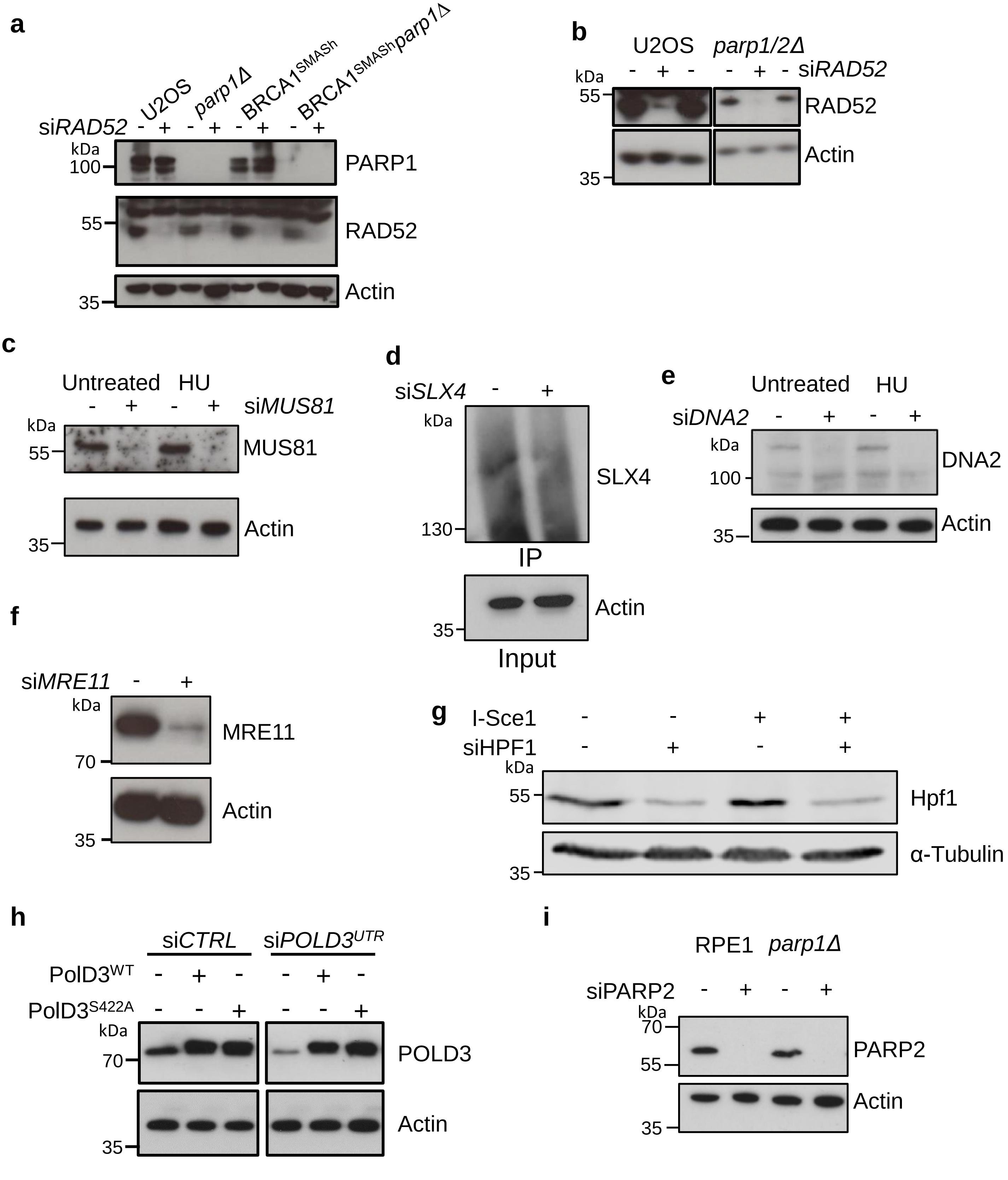

Supplementary Figure 3: Efficiency of siRNA depletion using the siRNAs employed in this study.

The indicted U2OS (a-h) or RPE-1 (i) derived cells were transfected with control (-) or gene specific siRNAs as indicated. Following preparation of whole cell extracts, western blotting was performed using the indicated antibodies. In (d) SLX4 was immunoprecipitated from whole cell extracts prior to western blotting. Immunoprecipitates (IP) are indicated. For loading controls inputs were probed with actin. Detection of Slx4 in inputs was not possible due to low expression levels and antibody sensitivity. All data in are representative of 3 independent experiments with the exception of (g) which is representative of 2 independent experiments.

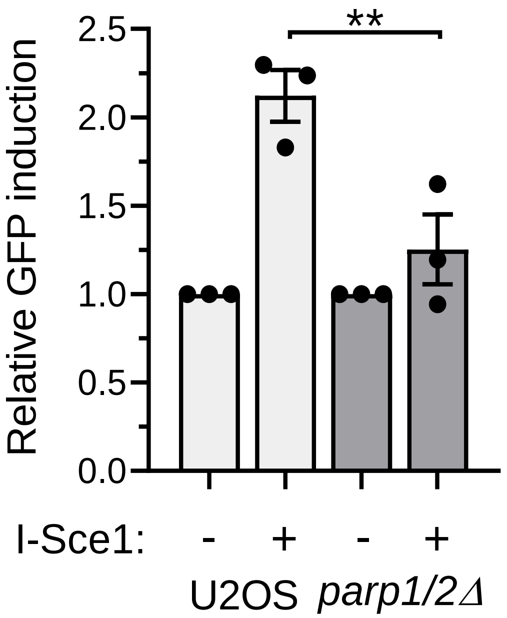

Supplementary Figure 4: Induction of GFP in response to I-Sce1 expression in U2OS or *parp1/2Δ* cells stably integrating with the *BIR GFP* reporter. GFP fluorescence was analysed by flow cytometry 72 h after transient *ISCE1* transfection. GFP induction was calculated relative to cells lacking I-Sce1 expression. Data are presented as mean values and error bars represent +/- SEM of three independent experiments. Statistical analysis was performed by one-way ANOVA with Tukey's test applied post-hoc. Statistical significance was calculated to a confidence of 95%, $p<0.05$ (*), 99%, $p<0.01$ (**) or 99.9%, $p<0.001$ (***).

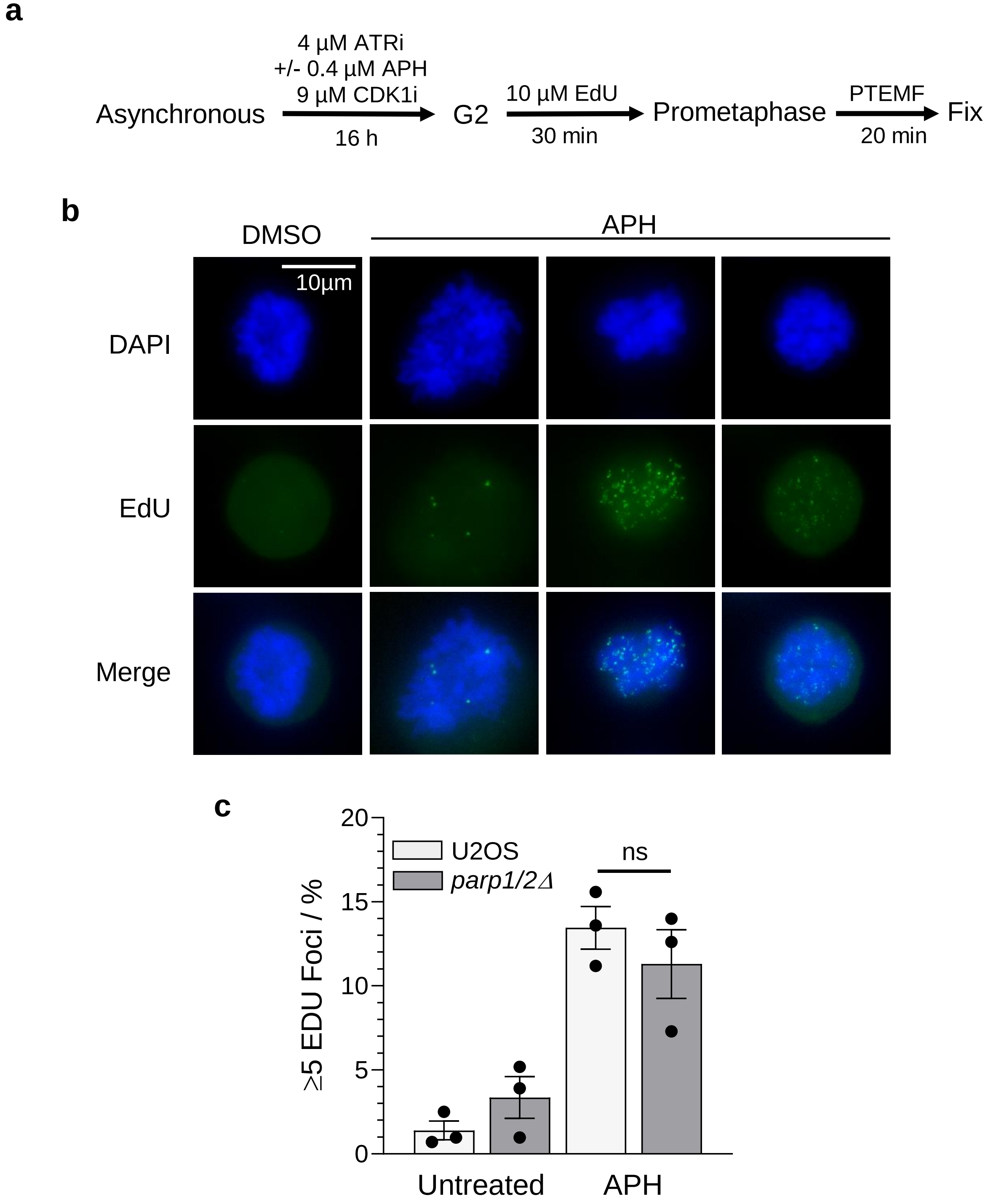

Supplementary Figure 5: The loss of PARP1/2 does not significantly affect mitotic DNA synthesis.

a. Asynchronous U2OS and *parp1/2Δ* cells were treated with 4µM VE-821(ATRi) and 0.4µM APH to induce replication stress in the presence of 9µM RO-3306 (CDK1i) to promote arrest at the G2/M boundary. Cells were released into prometaphase in the presence 10 µM EdU.
b. Representative images from 3 biological repeats showing detection of EdU incorporation by immunofluorescence in prometaphase cells. **c**. Quantification of the frequency of EdU-positive prometaphase cells. Data are presented as mean values and error bars represent +/- SEM of three biological repeats (n=3). Statistical significance was determined by a two-tailed Student's t-test.

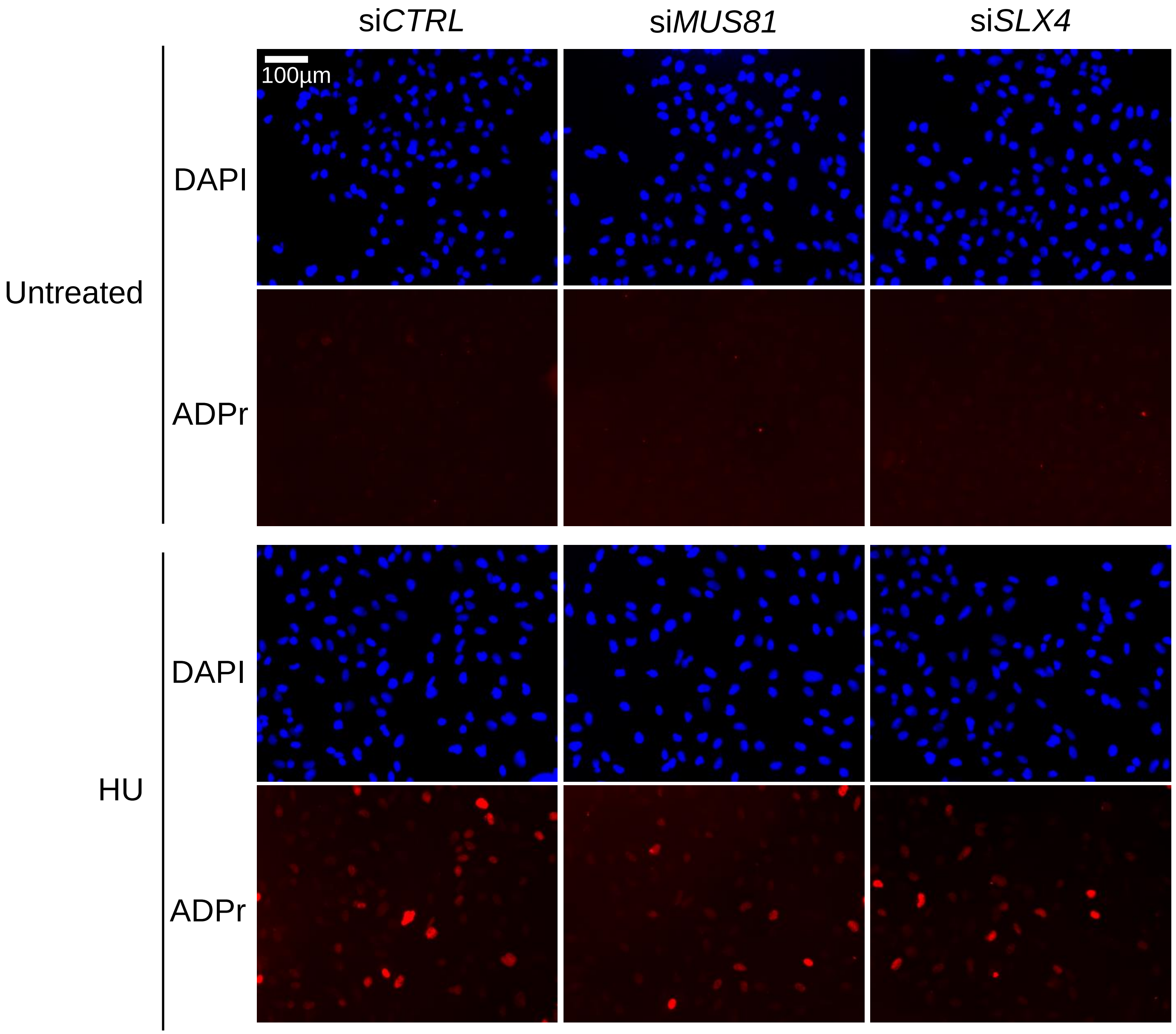

Supplementary Figure 6: HU-induced nuclear ADP-ribosylation signal in the absence of Mus81 and Slx4. a. Representative immunofluorescence images showing total nuclear ADPr staining using a reagent that detects mono- and poly-ADPr (ADPr). U2OS cells were transfected with control (siCTRL), MUS81 or SLX4 siRNA as indicated and left untreated or exposed to 24 hr 2mM HU as indicated. Data are representative of 3 independent experiments.

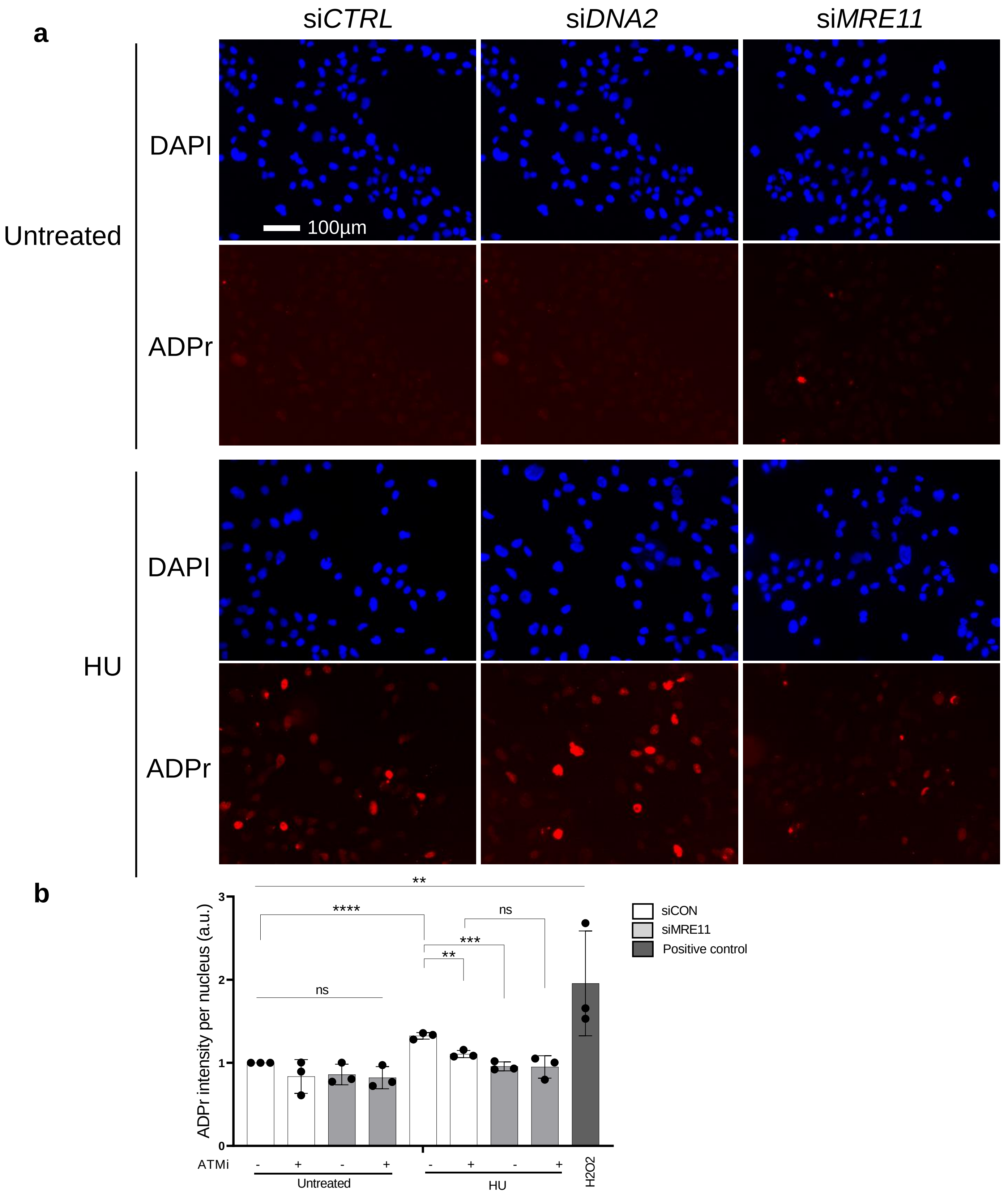

Supplementary Figure 7: HU-induced nuclear ADP-ribosylation signal in the absence of Dna2 and Mre11 and ATM activity. a. Representative immunofluorescence images from 3 independent experiments showing total nuclear ADPr staining using a reagent that detects mono- and poly-ADPr (ADPr). U2OS cells were transfected with control (siCTRL), DNA2 or MRE11 siRNA as indicated and left untreated or exposed to 24 hr 2mM HU **b**. Measurement of HU-induced ADPr intensity per nucleus (arbitrary units) using ADPr binding reagent in U2OS cells transfected with either siCTRL or siMRE11 siRNA. Cells were left untreated or exposed to 1µM ATM inhibitor (KU55933) and/or 2mM HU for 24 hr as indicated. H_2O_2 was used as a positive control for ADPr (10 minutes of 2mM). Error bars represent S.E.M from 3 biological repeats (n=3). Statistical significance was determined by a two-tailed Student's t-test, p<0.05 (*), 99%, p<0.01 (**) or 99.9%, p<0.001 (***). Otherwise, analyses were classified as not significant (ns).

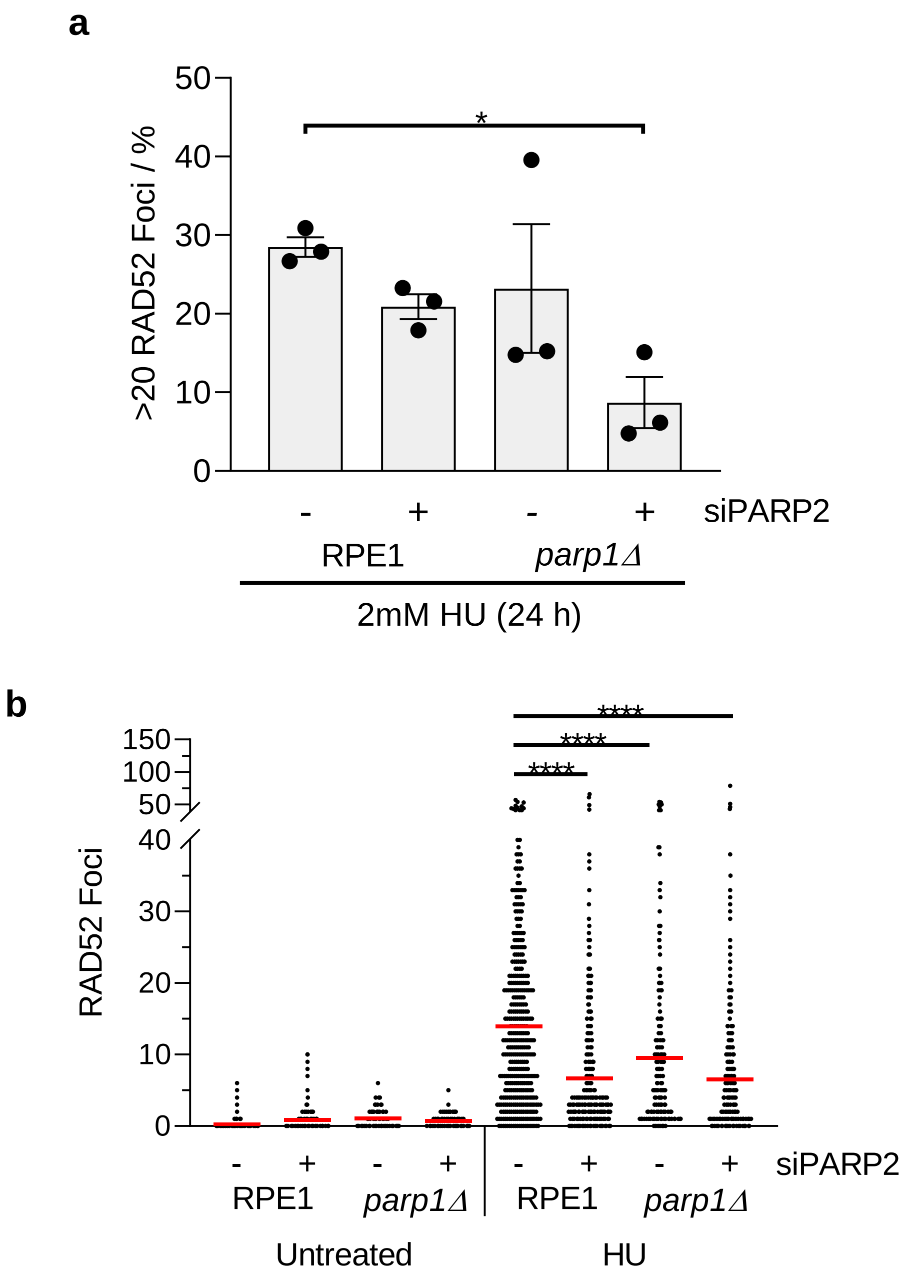

Supplementary Figure 8: HU-induced Rad52 foci formation is dependent on PARP1/2 in RPE-1 cells. Rad52 nuclear foci formation in response to 24 h HU treatment in parental (RPE1) and *PARP1*-disrupted (*parp1Δ*) RPE1 following siRNA-mediated *PARP2* depletion. **a**. Quantification of Rad52-positive nuclei. Error bars represent the S.E.M of three independent experiments. Statistical significance was determined following one-way ANOVA with Tukey's test applied post-hoc, $p<0.05$ (*). **b** . Distribution of RAD52 foci in RPE1 cells across three independent experiments. Red lines denote mean foci number. Statistical significance was determined by a Mann-Whitney test (two-sided), $p<0.0001$ (****).

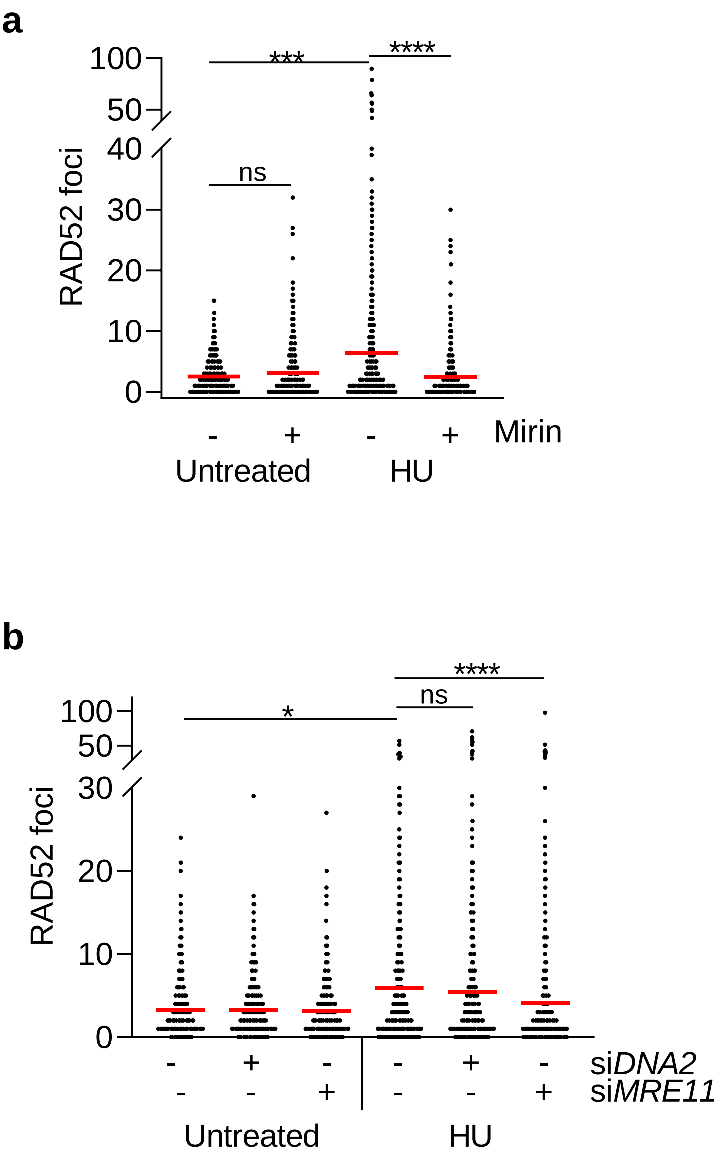

Supplementary Figure 9: HU-induced Rad52 foci formation is dependent on Mre11 nuclease activity. a. Quantification of Rad52 nuclear foci in U2OS cells treated with 2 mM HU for 24 h in the presence or absence of the Mre11 inhibitor, mirin (50 µM). **b** Quantification of Rad52 nuclear foci in U2OS cells treated with 2 mM HU for 24 h following depletion of the fork-resecting nucleases DNA2 and Mre11. At least 200 cells were scored per condition across three independent experiments. Red lines denote mean foci number. Statistical analysis was performed using a Mann-Whitney test (two-sided). Statistical significance was calculated to a confidence of 95%, $p<0.05$ (*); 99%, $p<0.01$ (**); 99.9%, $p<0.001$ (***); or 99.999%, $p<0.0001$ (****). Otherwise, analyses were classified as not significant (ns).

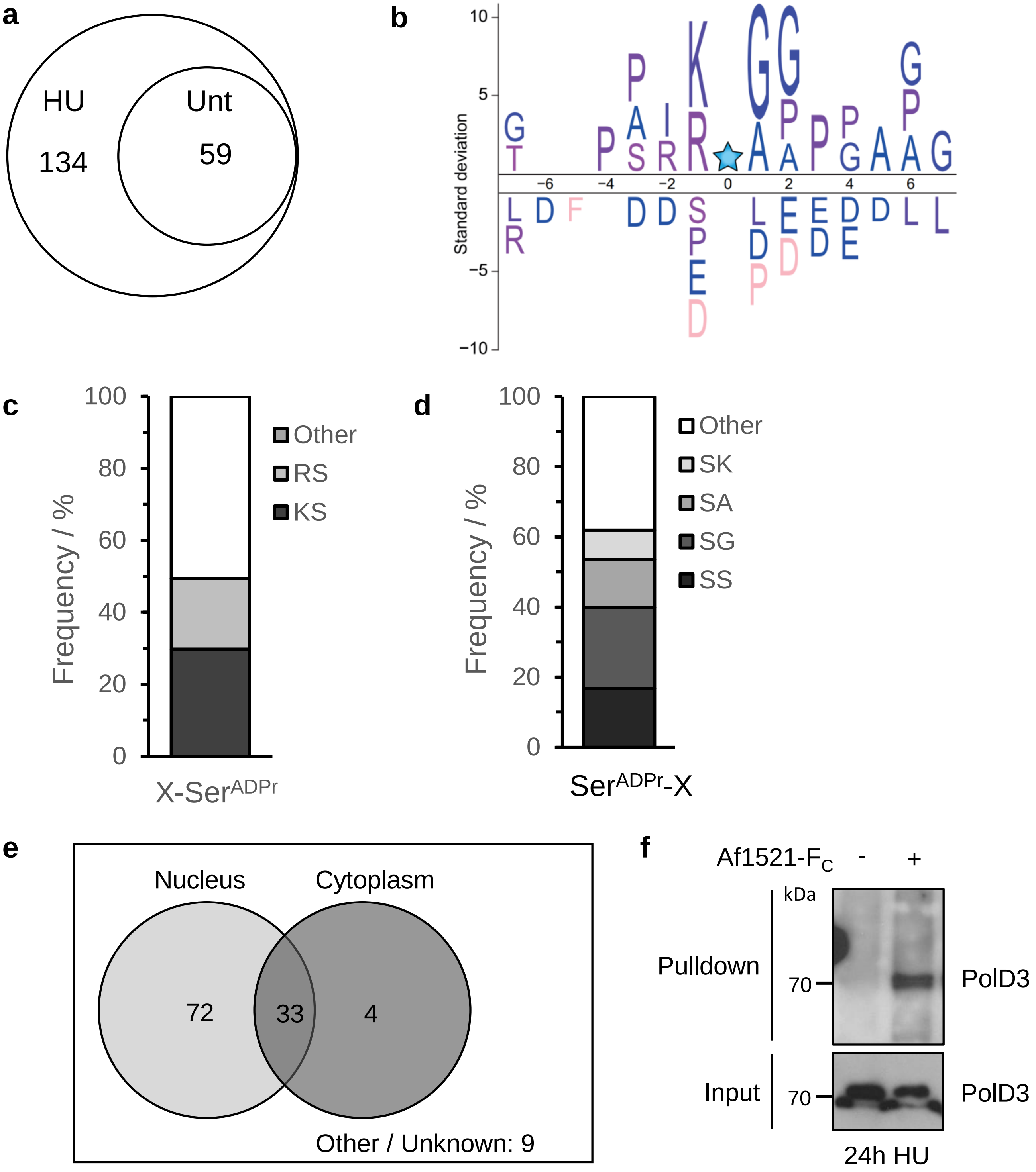

Supplementary Figure 10: Localisation and sequence context analyses of HU-induced ADP-ribosylation sites.

a. Venn diagram showing the distribution of 170 uniquely mapped ADPr sites within control (untreated) and experimental (HU) conditions.

b-d. IceLogo analysis showing the sequence context around identified ADPr sites (★; **b**). The frequency difference at each position is mapped for all amino acids which are enriched or depleted at each position. The sequence motif extracted from the IceLogo algorithm was selected at a significance level of $p<0.05$ using a two-sample t-test.compared against a *Homo sapiens* reference data set. Motif analyses surrounding Ser-localised ADPr sites highlighting common residues placed immediately to the N-terminus (**c**) or C-terminus (**d**).

e. Venn diagram showing the compartmentalisation of ADPr-modified proteins identified in this study.

f. Verification of PolD3 ADPr in response to 24 h HU. Following HU treatment of U2OS cells, ADPr proteins were affinity purified by PAN-ADP-ribose pulldown from U2OS whole cell lysates and the presence of PolD3 established by Western blot.

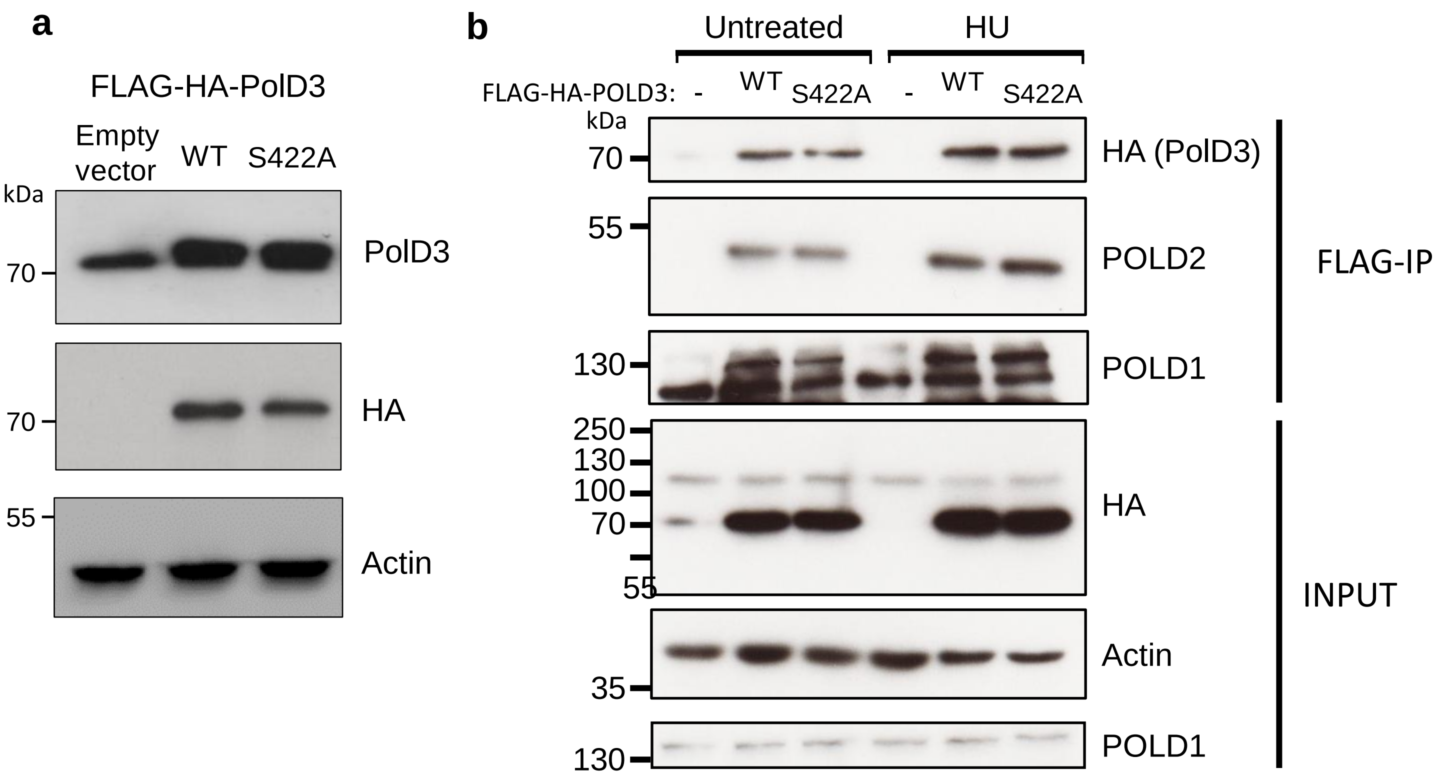

Supplementary Figure 11: Stable expression of the PolD3 ADP-ribosylation mutant and its interaction with PolD1 and PolD2.

a. Expression of FLAG-HA empty vector, FLAG-HA-PolD3 wild-type (WT) and FLAG-HA-PolD3^{S422A} ADP-ribosylation mutant (S422A) in U2OS cells. Whole cell extracts were analysed by western blotting performed with the indicated antibodies.

b. Co-immunoprecipitation of polymerase δ complex subunits following HU-induced replication stress. FLAG-HA-tagged wild-type (WT) or Ser-422-Ala mutant (S422A) FLAG-HA-PolD3 was immunoprecipitated from U2OS cells following exposure to 2mM HU for 24h. FLAG-HA-PolD3 and associated PolD1 and PolD2 levels were assessed by western blotting performed with the indicated antibodies. Images are representative of 3 independent experiments.

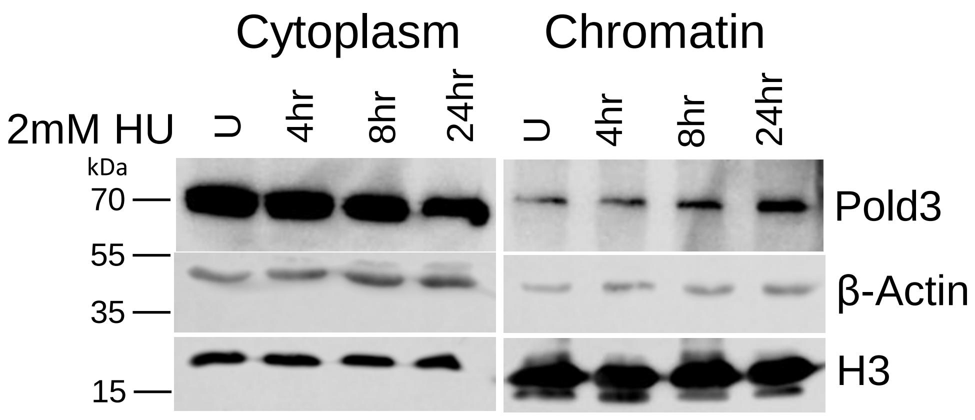

Supplementary Figure 12: PolD3 assembly into chromatin in response to HU. Assessment of PolD3 Chromatin-bound levels in U2OS WT cells, following exposure to 2 mM HU for the times indicated. Cytoplasm and Chromatin biochemical fractionated extracts were analysed by SDS-PAGE and western blotting performed using the indicated antibodies. Data are representative of 3 independent experiments.

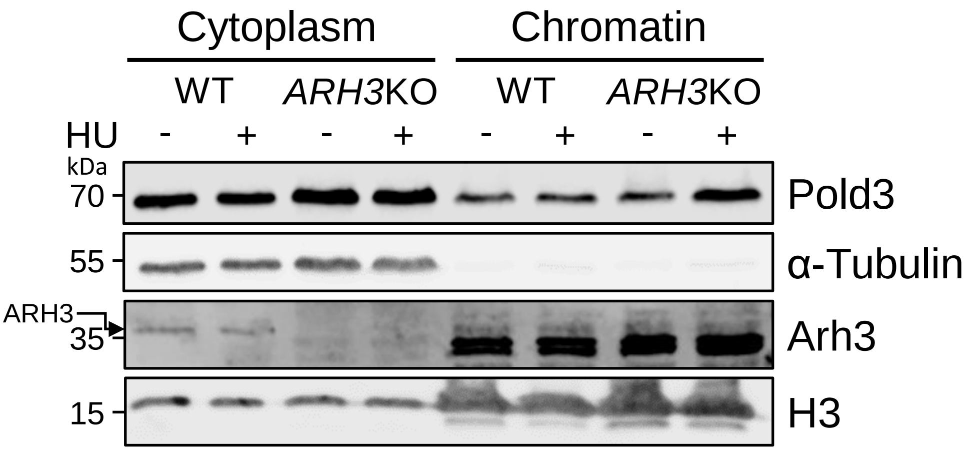

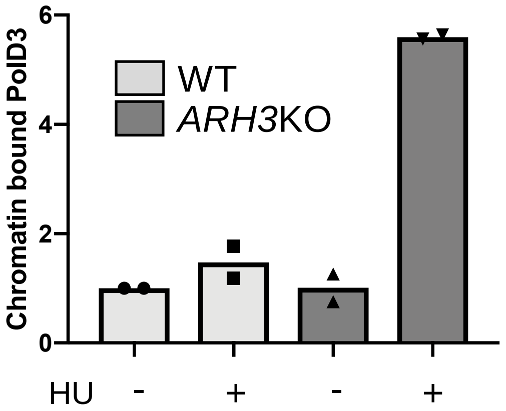

Supplementary Figure 13: Assessment of PolD3 Chromatin-bound levels in ARH3 knockout (*ARH3*KO) U2OS cells following exposure to 2 mM HU for 24 h. Cytoplasm and Chromatin biochemical fractionated extracts were analysed by SDS-PAGE and western blotting using the indicated antibodies. Quantification of ARH3 chromatin enrichment levels relative to the non-specific band in ARH3 blots is represented in the lower panel. The mean is represented from 2 biological repeats.

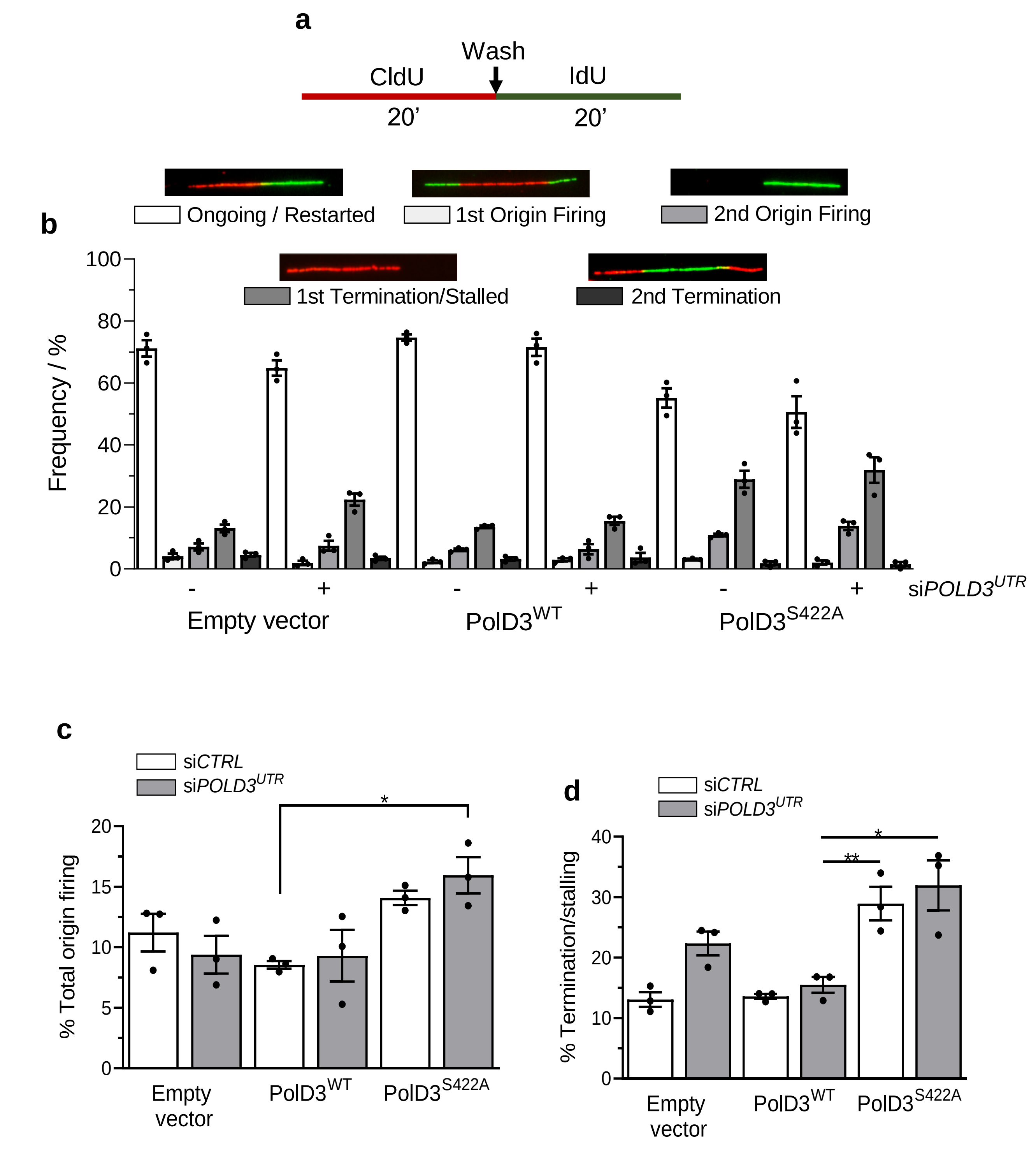

Supplementary Figure 14: DNA fibre analysis showing effects of PolD3^{S422A} mutant expression on unperturbed replication dynamics.

a. CldU-IdU labelling scheme. **b**. Relative frequencies of different replication structures identified in U2OS cells expressing siRNA-resistant PolD3^{WT} or the PolD3^{S422A} ADPr mutant either in the absence or presence of siRNA depletion of endogenous PolD3 as indicated. DNA fibre classifications describe continuously replicating forks (ongoing) as well as origin firing and termination events. Representative images of DNA fibres are shown above each category within the legend. **c** and **d**. The frequency of total origin firing and 1st label termination/stalling events from the same dataset are shown in (**c**) and (**d**), respectively. Error bars represent S.E.M from 3 biological repeats (n=3). Statistical analysis was performed by one-way ANOVA with Tukey’s test applied post-hoc, $p<0.05$ (*), $p<0.01$ (**).
