# Supplementary material for: Regulation of Rad52-dependent replication fork recovery through serine ADP-ribosylation of PolD3
Source: Nat Commun. 2023 Jul 18;14:4310. doi: 10.1038/s41467-023-40071-w (PMC10354178; doi:10.1038/s41467-023-40071-w)
Supplement: Supplementary file 3 — Description of Additional Supplementary Files [file 41467_2023_40071_MOESM3_ESM.pdf]

Description of Additional Supplementary Files

File Name: Supplementary Data 1
Description: Overview of ADPr sites. A list of ADPr sites identified in this study either in untreated cells (Con) or at times following exposure to HU as indicated.

File Name: Supplementary Data 2
Description: List of antibodies used in this study.
