# Supplementary material for: Regulation of Rad52-dependent replication fork recovery through serine ADP-ribosylation of PolD3
Source: Nat Commun. 2023 Jul 18;14:4310. doi: 10.1038/s41467-023-40071-w (PMC10354178; doi:10.1038/s41467-023-40071-w)
Supplement: Supplementary file 6 — Reporting Summary [file 41467_2023_40071_MOESM6_ESM.pdf]

nature portfolio

Corresponding author(s): N D Lakin

Last updated by author(s): Jul 29, 2022

Reporting Summary

Nature Portfolio wishes to improve the reproducibility of the work that we publish. This form provides structure for consistency and transparency in reporting. For further information on Nature Portfolio policies, see our Editorial Policies and the Editorial Policy Checklist.

Statistics

For all statistical analyses, confirm that the following items are present in the figure legend, table legend, main text, or Methods section.

n/a	Confirmed	
☐	☒	The exact sample size (*n*) for each experimental group/condition, given as a discrete number and unit of measurement
☐	☒	A statement on whether measurements were taken from distinct samples or whether the same sample was measured repeatedly
☐	☒	The statistical test(s) used AND whether they are one- or two-sided<br>*Only common tests should be described solely by name; describe more complex techniques in the Methods section.*
☒	☐	A description of all covariates tested
☒	☐	A description of any assumptions or corrections, such as tests of normality and adjustment for multiple comparisons
☐	☒	A full description of the statistical parameters including central tendency (e.g. means) or other basic estimates (e.g. regression coefficient) AND variation (e.g. standard deviation) or associated estimates of uncertainty (e.g. confidence intervals)
☐	☒	For null hypothesis testing, the test statistic (e.g. *F*, *t*, *r*) with confidence intervals, effect sizes, degrees of freedom and *P* value noted<br>*Give P values as exact values whenever suitable.*
☒	☐	For Bayesian analysis, information on the choice of priors and Markov chain Monte Carlo settings
☒	☐	For hierarchical and complex designs, identification of the appropriate level for tests and full reporting of outcomes
☒	☐	Estimates of effect sizes (e.g. Cohen's *d*, Pearson's *r*), indicating how they were calculated

Our web collection on statistics for biologists contains articles on many of the points above.

Software and code

Policy information about availability of computer code

Data collection	Image Studio Lite version 5.2 for western blot quantification
Data analysis	ImageJ 1.53q, Microsoft excel 2016, Graphpad Prism 9, MaxQuant version 1.5.3.30

For manuscripts utilizing custom algorithms or software that are central to the research but not yet described in published literature, software must be made available to editors and reviewers. We strongly encourage code deposition in a community repository (e.g. GitHub). See the Nature Portfolio guidelines for submitting code & software for further information.

Data

Policy information about availability of data

All manuscripts must include a data availability statement. This statement should provide the following information, where applicable:

- Accession codes, unique identifiers, or web links for publicly available datasets
- A description of any restrictions on data availability
- For clinical datasets or third party data, please ensure that the statement adheres to our policy

All data generated or analysed during this study are included in this published article (and its supplementary information files), or available from the corresponding author on request.

The mass spectrometry proteomics data have been deposited to the ProteomeXchange Consortium via the PRIDE partner repository with the dataset identifier PXD035661.

Human research participants

Policy information about studies involving human research participants and Sex and Gender in Research.

Reporting on sex and gender	Not applicable
Population characteristics	Not applicable
Recruitment	Not applicable
Ethics oversight	Not applicable

Note that full information on the approval of the study protocol must also be provided in the manuscript.

Field-specific reporting

Please select the one below that is the best fit for your research. If you are not sure, read the appropriate sections before making your selection.

☒ Life sciences ☐ Behavioural & social sciences ☐ Ecological, evolutionary & environmental sciences

For a reference copy of the document with all sections, see nature.com/documents/nr-reporting-summary-flat.pdf

Life sciences study design

All studies must disclose on these points even when the disclosure is negative.

Sample size	As is standard practice for the field, statistical analysis were performed on data derived from at least 3 biological replicates.
Data exclusions	No data are excluded form the analyses.
Replication	Data is derived from at least 3 biological repeats. All repeats were successful and where possible data used to perform statistical analysis.
Randomization	Samples were allocated into group depending on cell treatments as described in the manuscript. Due to the relatively small group numbers randomization was not employed during these experiments.
Blinding	Where possible (e.g. immunoflourescence), samples were scored blind. In certain experiments (e.g. western blotting) it was not possible to anonymise the data.

Reporting for specific materials, systems and methods

We require information from authors about some types of materials, experimental systems and methods used in many studies. Here, indicate whether each material, system or method listed is relevant to your study. If you are not sure if a list item applies to your research, read the appropriate section before selecting a response.

Materials & experimental systems

n/a	Involved in the study	
☐	☒	Antibodies
☐	☒	Eukaryotic cell lines
☒	☐	Palaeontology and archaeology
☒	☐	Animals and other organisms
☒	☐	Clinical data
☒	☐	Dual use research of concern

Methods

n/a	Involved in the study	
☒	☐	ChIP-seq
☒	☐	Flow cytometry
☒	☐	MRI-based neuroimaging

Antibodies

Antibodies used

anti-BRCA1 (Millipore 07-434)
anti-actin (Santa-Cruz® sc-1615)
anti-H2AX-P (Abcam® ab11174)
anti-Rad52: For foci a gift from Jiri Lukas (Ochs eta al. (2016), Nat. Struct Mol Biol 23:714) for western blotting GeneTex #GTX70301

anti-H3 (Abcam® ab12079)
anti-flag (SIGMA® F1804 and F7425)
anti-PAN-ADPr (Merk® MABE1016)
anti-PolD3 (Abnova H00010714-M01)
anti-HA (Cell Signalling Technology #3724)
IRDye 800 CW-Secondary (Li-cor 926-32213)
IRDye 700 CW-Secondary (Li-cor 926-32210)
anti-HPF1: A gift from Ivan Ahel (Bonfiglio JJ, et al. Serine ADP-Ribosylation Depends on HPF1. Molecular cell 65, 932-940 e936 [2017]).
anti-ARH3 (Santa Cruz sc-374162)

Validation

With the exception of anti-Rad52 and anti-BRCA1, all antibodies have previously been employed and validated in the laboratory (see Ronson et al. (2018), Nat Commun 9:746; Brustel et al. (2022), Nat Commun 13:185; Couto et al. (2011), J Cell Biol, 194:367-375; Rakhimova et al., Sci Rep (2017), 7:43750-43761).

For anti-Rad52 see Ochs et al. (2016), Nat. Struct Mol Biol 23:714 and https://www.genetex.com/Product/Detail/Rad52-antibody-5H9/GTX70301

For commercial antibodies see:
anti-BRCA1; Figure 1 and https://www.merckmillipore.com/GB/en/product/Anti-BRCA1-Antibody,MM_NF-07-434?ReferrerURL=https%3A%2F%2Fwww.google.com%2F
anti-Actin;https://www.scbt.com/p/beta-actin-antibody-c4?gclid=EAIaIQobChMIxpHD-OeY-QIViK3tCh1r2QdtEAAYASAAEgKiz_D_BwE
anti-H2AX-P; https://www.abcam.com/gamma-h2ax-phospho-s139-antibody-ab11174.html
anti-H3; https://www.abcam.com/histone-h3-antibody-chip-grade-ab12079.html
anti-flag; https://www.sigmaaldrich.com/GB/en/product/sigma/f1804?gclid=EAIaIQobChMIqqfMu-iY-QIVy7TtCh1Doge8EAAYASAAEgLJ_PD_BwE and https://www.sigmaaldrich.com/GB/en/product/sigma/f7425
anti-PAN-ADPr; https://www.sigmaaldrich.com/GB/en/product/mm/mabe1016?gclid=EAIaIQobChMIzID-kemY-QIVBO3tCh1jkQPCEAAYASAAEgLcavD_BwE
anti-PolD3; http://www.abnova.com/products/products_detail.asp?catalog_id=H00010714-M01
anti-HA; https://www.cellsignal.co.uk/products/primary-antibodies/ha-tag-c29f4-rabbit-mab/3724
anti-BrdU; https://www.abcam.com/brdu-antibody-bu175-icr1-proliferation-marker-ab6326.html and https://www.bdbiosciences.com/en-gb/products/reagents/flow-cytometry-reagents/clinical-discovery-research/single-color-antibodies-ruo-gmp/purified-mouse-anti-brdu.347580
anti-HPF1; https://www.novusbio.com/products/hpf1-antibody_nbp1-93973
anti-ARH3; https://www.scbt.com/p/arh3-antibody-a-7
anti-Mono-ADPr (AbD33205ad); https://www.bio-rad-antibodies.com/monoclonal/protein-peptide-tag-mono-adp-ribose-antibody-abd33205ad-tza021.html?f=purified

Eukaryotic cell lines

Policy information about cell lines and Sex and Gender in Research

Cell line source(s)

All cell lines have been described previously (Ronson et al. (2018), Nat Commun 9:746), or are derived from U2OS cells used in this study. An exception is HEK293T cells for virus production which were from Lakin lab stocks.

Authentication

We did not manipulate HEK293T cells and so have not validated them. Other cell lines were authenticated previously (Ronson et al. (2018), Nat Commun 9:746). For derivatives of these cells generated during this study, the genotype was verified by PCR amplification and sequencing of the relevant loci, and protein levels established by western blotting with the appropriate antibodies.

Mycoplasma contamination

All cell lines tested negative for mycoplasma infection.

Commonly misidentified lines
(See ICLAC register)

No commonly misidentified lines were used in this study.
